# Obtention of solar cell parameters, through convergence of iterative cycles. Part 1: Theoretical analysis and cycles proposal

**DOI:** 10.1016/j.heliyon.2022.e10551

**Published:** 2022-09-15

**Authors:** Victor-Tapio Rangel-Kuoppa

**Affiliations:** Department of Physics, Lancaster University, Lancaster, United Kingdom

**Keywords:** Solar cell, Light current, Saturation current, Series resistance, Shunt resistance, Ideality factor, Parameter extraction

## Abstract

In this Part 1 of this series of articles, two iterative cycles are proposed to accurately determine the shunt resistance ($R_{sh}$), the series resistance ($R_{s}$), the ideality factor (*n*), the light current ($I_{\mathrm{lig}}$), and the saturation current ($I_{\mathrm{sat}}$) of solar cells, within the one diode model. First, $R_{s}$ and *n* are obtained linearly fitting $\frac{\partial V}{\partial lnI^{\prime }}$*vs.*$I^{\prime }$, where $I^{\prime }$ is a new defined current $I^{\prime }=I+I_{sat}+I_{lig}-\frac{V-IR_{s}}{R_{sh}}+\frac{nkT}{R_{sh}}$. Then, $R_{sh}$ and $I_{\mathrm{sat}}$ are obtained using Procedure A and B proposed in [2]. Once these four solar cell parameters are obtained, a correction to $I_{\mathrm{lig}}$ is deduced and applied. The deduction of these five solar cell parameters is reused to recalculate $I^{\prime }$ and the iterative cycles are redone till some convergence criteria is achieved. The accuracy and number of cycles necessary to achieve reasonable results are tested and discussed on ideal (noiseless) current voltage (*IV*) curves with measured points per voltage of $P_{V}=11$, 21, 51 and 101 $\frac{\text{measured points}}{V}$. These two cycles are compared with two different common parameter extraction methods. The results given in this Part 1 are used in Part 2 to calculate the five solar cell parameters of *IV* curves found in the literature.

## 1 Introduction

The world ecological problems are well known, due to the mass consumption of fossil fuels in the last centuries. Concomitantly, humanity energy consumption continues increasing every year, and it is foretold that it will increase from the current value of 10 TW to around 30 TW by the year 2050. This increase in energy consumption, together with the ecological problems, impel humanity to find and enact nature-friendly methods to produce energy, i.e., reducing significantly the CO_2_ and other carbon related gases emission. Solar cells have shown to be suitable candidates to accomplish this, as they produce cheap energy in an ecological manner [1].

Several models can be found in the literature to describe the solar cell performance, but it is the one-diode solar cell model which is the most used, due to its simplicity (see equation (1) and Fig. 1 in [2]). For this solar cell model, the parameters are the shunt resistance ($R_{sh}$), the series resistance ($R_{s}$), the light current ($I_{\mathrm{lig}}$), the ideality factor (*n*), and the saturation current ($I_{\mathrm{sat}}$), according to Fig. 1 in [2]. Several types of measurements are performed on the solar cells to obtain these parameters. The current voltage (*IV*) measurement is one of the most widely used techniques, both in darkness and illumination, to extract these solar cell parameters [3], [4].

The accurate determination of $R_{sh}$, $R_{s}$, $I_{\mathrm{lig}}$, $I_{\mathrm{sat}}$ and *n* is of utterly importance. Scientifically, they provide valuable information of any physical phenomena occurring within the solar cell, which in turns helps to technologically improve them, resulting in suitable commercial products. For example, a low value of $R_{sh}$ is a signal of poor material manufacturing quality (like crystal defects and unintentional impurities), providing alternative current paths to the generated electron-hole pairs [5]. On the contrary, a large value of $R_{s}$ reveals bad ohmic contacts and/or high material resistance [6], [7]. On both cases, they have the pernicious effect to reduce the efficiency of the solar cell [5], [6], [7]. Regarding $I_{\mathrm{sat}}$ and *n*, they are an indication of the solar cell quality [8]. $I_{\mathrm{sat}}$ represents the sum of all the recombination mechanisms inside the emitter, such as Auger, Schockley-Read-Hall (SRH), contact and surface mechanisms [8]. $I_{\mathrm{sat}}$ is also affected by band-gap narrowing due to heavy doping effects [8]. In the case of *n*, a value of 1 is due to minority carrier diffusion, while a value smaller or equal to 2 is evidence that the charge carrier generation/recombination occurs within the depletion region. Values larger than 2 could be due to shunt resistance effects and/or non-uniformities in the distribution of recombination centres [9]. Finally, $I_{\mathrm{lig}}$ (also known as the photocurrent) depends on the donor and acceptor densities, as on the life times of the charge carriers [10]. Several articles on their impact on the solar cell performance have been reported [5], [6], [7], [8], [9], [10], [11], [12].

A brief discussion of the available analysis techniques to determine $R_{sh}$, $R_{s}$, $I_{\mathrm{lig}}$, $I_{\mathrm{sat}}$ and *n* for a single *IV*, independently of illumination conditions, can be found in the introduction of [13]. Briefly, only a handful of techniques can be used to a single *IV* curves, independent of illumination conditions and approximations. One of them is the Ortiz-Conde *et al.* method [14], but its accuracy is very sensitive to noise and number of measured points [13], [15]. Other techniques are Procedure A and Procedure B proposed in [2], [16]. Finally, other methods are based in Monte Carlo simulations, artificial neuronal networks, non-linear least-squares method, exponential model, or ab initio calculations [17], [18], [19], [20], [21], [22], [23], [24], [25], [26]. A review of the limitations and assumption validity of these main techniques can be found in [27]. This explains the intention of this series of articles. To propose two iterative cycles to determine more accurately the solar cell parameters, within the one-diode solar cell model.

This article is divided into the following sections. Following this brief introduction, in Section 2 the theoretical analysis and the proposal of the iterative cycles, is given. At the same time, the description of the programs CycleB, CycleAroot and CycleAmanual is provided. Section 3 describes that a density of points of $P_{V}=11\frac{measured\;points}{V}$ in the *IV* curves is not enough to obtain convergence using program CycleAmanual, while this value of $P_{V}$ is enough to obtain convergence using program CycleB. Something similar happens with a value of $P_{V}=21\frac{measured\;points}{V}$, as it is shown in Section 4. In both Sections 3 and 4, the problems of program CycleAroot to obtain the right value of $I_{sat}$ to achieve $m_{Rsh}=0$, are described. It is concluded that the right value of $I_{sat}$ to achieve $m_{Rsh}=0$ should be obtained manually, as it is done in program CycleAmanual. Section 5 is next, where a value of $P_{V}=51\frac{measured\;points}{V}$ is used and discussed. In this case, and with larger values of $P_{V}$, convergence is obtained using either program CycleAmanual or program CycleB. In Section 6 a similar analysis is done, but considering a value of $P_{V}=101\frac{measured\;points}{V}$. In Section 7, the application of the Ortiz-Conde *et al.* technique [14] and the Zhang *et al.* technique [28] is done on the *IV* curves, for comparison purposes. Notice that this application is done on the ideal (noiseless) *IV* curves, to simplify comparison, as it has been shown that unrealistic parameters can be obtained, in case the *IV* curves have noise [13], [15], using the Ortiz-Conde *et al.* technique [14]. Discussion is given in Section 8 and finally, conclusions are given in Section 9.

## 2 Theoretical analysis

For completeness purposes, and as it will be used in the discussion below, the *IV* curve of a solar cell, within the one-diode model (equation (1) in [2]) is reproduced next:

(1) $$I=I_{sat}\left(e^{\frac{V-IR_{s}}{nkT}}-1\right)+\frac{V-IR_{s}}{R_{sh}}-I_{lig}$$

In their proposal of two procedures to obtain $R_{sh}$ and $I_{\mathrm{sat}}$ [2], [16], Rangel-Kuoppa *et al.* first extended the Cheung method [29], [30], [31], which was originally proposed for Schottky contacts, to use it in equation (1), obtaining an upper limit for $I_{\mathrm{sat}}$. According to the discussion in Section 2 in [2], the application of the Cheung method is achieved assuming $R_{sh}\to \infty$ and $e^{\frac{V-IR_{s}}{nkT}}\gg 1$. This allows to rewrite equation (1) as

(2) $$\frac{\partial V}{\partial lnI}=nkT+R_{s}I$$

The linear fit of $\frac{\partial V}{\partial lnI}$ *vs. I* yields $R_{s}$ and *n*, while $I_{\mathrm{lig}}$ is approximated to the short-circuit current ($I_{sc}$), i.e., $I_{lig}≅-I_{sc}$. Once this is achieved, values for $R_{sh}$ and $I_{\mathrm{sat}}$ were obtained, using the Procedures A and B proposed in [2] (see Section 2 in [2]). Using these procedures, the solar cell parameters were obtained, and comparison with other two popular procedures, namely the Zhang *et al.* [28], and the Ortiz-Conde *et al.* [14], more accurate determination was achieved (see Section 4 in [2] and Sections 4 and 5 in [16]).

Once $R_{sh}$ and $I_{\mathrm{sat}}$ were obtained, it would be very convenient, if they could be considered again in the application of equation (2). To achieve this, the following analysis is done.

If the $\frac{\partial }{\partial I}$ in equation (1) is done, the following expression is obtained:

(3) $$nkT+R_{s}\left(I+I_{sat}+I_{lig}-\frac{V-IR_{s}}{R_{sh}}+\frac{nkT}{R_{sh}}\right)\;=\left(I+I_{sat}+I_{lig}-\frac{V-IR_{s}}{R_{sh}}+\frac{nkT}{R_{sh}}\right)\frac{\partial V}{\partial I}$$

Defining a new current $I^{\prime }$

(4) $$I^{\prime }=I+I_{sat}+I_{lig}-\frac{V-IR_{s}}{R_{sh}}+\frac{nkT}{R_{sh}}$$

equation (3) can be rewritten as

(5) $$nkT+R_{s}\left(I^{\prime }\right)=\left(I^{\prime }\right)\frac{\partial V}{\partial I}$$

At the same time, it is well known that

(6) $$\frac{\partial V}{\partial lnI^{\prime }}=\frac{\partial V}{\partial I^{\prime }}\frac{\partial I^{\prime }}{\partial lnI^{\prime }}=I^{\prime }\frac{\partial V}{\partial I^{\prime }}$$

Then, in order to write equation (5) as equation (2), to apply the same linear fit to obtain $R_{s}$ and *n*, it is necessary to prove that $\frac{\partial V}{\partial I^{\prime }}=\frac{\partial V}{\partial I}$. This is done next. On one hand

(7) $$\frac{\partial V}{\partial I}=\frac{\partial V}{\partial I^{\prime }}\frac{\partial I^{\prime }}{\partial I}$$

At the same time, using equation (4), $\frac{\partial I^{\prime }}{\partial I}$ can be expressed as

(8) $$\frac{\partial I^{\prime }}{\partial I}=1+\frac{R_{s}}{R_{sh}}-\frac{1}{R_{sh}}\frac{\partial V}{\partial I}$$

It is known that the following approximation is valid for large voltages [32]

(9) $$\frac{\partial V}{\partial I}∼R_{s}$$

Introducing equation (9) into equation (8) yields

(10) $$\frac{\partial I^{\prime }}{\partial I}=1$$

And then equation (7) becomes

(11) $$\frac{\partial V}{\partial I}=\frac{\partial V}{\partial I^{\prime }}$$

Introducing equation (11) in equation (5) and using equation (6), then equation (4) becomes

(12) $$\frac{\partial V}{\partial lnI^{\prime }}=nkT+R_{s}\left(I^{\prime }\right)$$

which is mathematical indistinguishable from equation (2), and then the linear fit of $\frac{\partial V}{\partial lnI^{\prime }}$ versus $I^{\prime }$ yields again $R_{s}$ and *n*, in this case using the definition of $I^{\prime }$ (equation (4)), i.e., after considering the values of $R_{sh}$, $R_{s}$, $I_{\mathrm{lig}}$, $I_{\mathrm{sat}}$ and *n* determined the first time using the linear fit of equation (2) and Procedures A or B in [2].

Simultaneously, once an estimation of $R_{sh}$, $R_{s}$, $I_{\mathrm{lig}}$, $I_{\mathrm{sat}}$ and *n* were obtained, it would also be convenient if they could be used to refine the estimation of $I_{lig}$ as $-I_{sc}$, i.e., $I_{lig}≅-I_{sc}$.

Using the approximation at low voltages:

(13) $$e^{\frac{V-IR_{s}}{nkT}}∼1+\frac{V-IR_{s}}{nkT}$$

equation (1) can be rewritten as

(14) $$I=I_{sat}\left(\frac{V-IR_{s}}{nkT}\right)+\frac{V-IR_{s}}{R_{sh}}-I_{lig}$$

Evaluating equation (14) at V=0, then $I\left(V=0\right)=I_{sc}$, and solving for $I_{lig}$ the following expression is obtained

(15) $$I_{lig}≅-I_{sc}\left(1+\frac{R_{s}}{R_{sh}}+\frac{I_{sat}R_{s}}{nkT}\right)$$

As they will be needed in the proposal done next, the equations of $R_{sh}$ and $I_{sat}$ (equation s (9) and (10) in [2]) are reproduced:

(16) $$R_{sh}=\frac{V-IR_{s}}{I+I_{lig}-I_{sat}\left(e^{\frac{V-IR_{s}}{nkT}}-1\right)}$$

(17) $$I_{sat}=\frac{I+I_{lig}-\frac{V-IR_{s}}{R_{sh}}}{e^{\frac{V-IR_{s}}{nkT}}-1}$$

**Figure 1:**
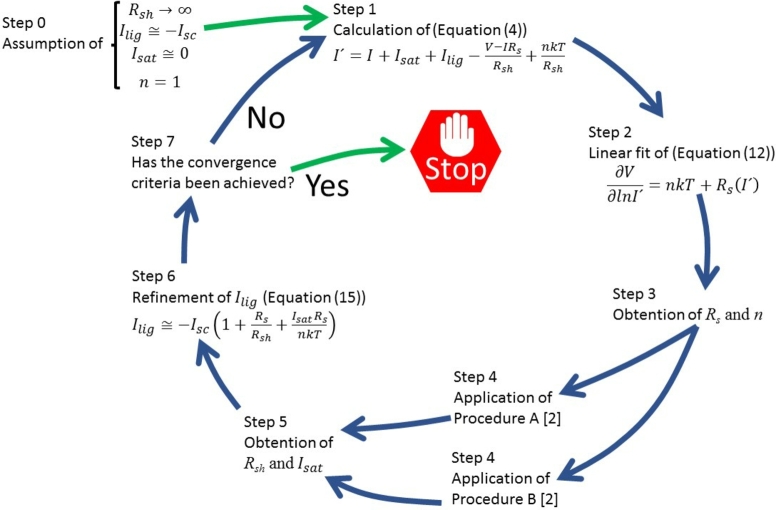
Schematic view of the proposed iterative cycles.

The former discussion, suggests that the following cycles could be implemented. First, Cheung *et al.* method is applied in equation (2), assuming $R_{sh}\to \infty$, $I_{lig}≅-I_{sc}$ and $I_{sat}≅0$. This provides $R_{s}$ and *n*. Second, either Procedure A or B in [16] is applied to obtain $R_{sh}$ and $I_{sat}$. Once they are obtained, the refinement of $I_{lig}$ using equation (15) is calculated, and using the solar cell parameters so far obtained, the new $I^{\prime }$ proposed in equation (4) is calculated, and the Cheung method is used again, this time using $I^{\prime }$, to obtain new $R_{s}$ and *n*, which are again used one more time in Procedure A or B in [16] to obtain new $R_{sh}$ and $I_{sat}$. The cycle continues till some convergence criteria is achieved. This is schematically shown in Fig. 1.

The proposed cycles were programmed. In case Procedure A (Procedure B) is used in the cycle, then the cycle is called CycleA (CycleB).

For both programs, when the cycles are started, the linear fit of equation (12) is done, calculating $I^{\prime }$ in equation (4). For the first cycle, $I^{\prime }$ is calculated assuming the following approximations: $I_{lig}≅-I_{sc}$, n=1, $I_{sat}=0$ and $R_{sh}=10^{30}\;\Omega$. This is equivalent to transform equation (12) to equation (6) in [2]. Once the linear fit of equation (12) was done and the first estimations of *n* and $R_{s}$ were obtained, then either Procedure A or B was used. This is discussed next.

The nature of equation (17) causes that, when Procedure B is used, the plot of $I_{sat}$ decreases from positive values close to V=0 if $R_{sh}$ is below the correct value of $R_{sh}$, becoming negative for values of $R_{sh}$ larger than the correct value of $R_{sh}$ (see for example Fig. 7 in [2]). This means that the slope of the linear fit of equation (17) ($m_{Isat}$), is positive (negative) for values of $R_{sh}$ lower (larger) than the correct value of $R_{sh}$. Then, the program for CycleB was done, such that the user can indicate an initial and final $R_{sh}$ and the number of $R_{sh}$ points to be tested in the interval. The program calculated each $m_{Isat}$ of the linear fitting of equation (17) for each $R_{sh}$ in the defined interval and plot as function of $R_{sh}$. Varying the $R_{sh}$ interval, the user finds the optimal value of $R_{sh}$ that causes $m_{Isat}=0$. At the same time, as it is discussed in Section 3.2 in [2], equation (17) has a negative resonance when V→0 and it is necessary to avoid it, skipping some measured points close to V=0. The program was implemented such that user can indicated how many of these points are skipped when calculating $m_{Isat}$ for each $R_{sh}$. A video example (named CycleB) of the use of program CycleB can be found in the supplementary material [33].

A similar idea was attempted using Procedure A. However, equation (16) does not show the same nature as the one just described for equation (17), *i.e*., that a single root is easily observed when plotting $m_{Rsh}$ *vs.* $I_{sat}$. This can be seen, for example, in Fig. 5 in [2]. When varying the value of $I_{sat}$ to find the optimal value such that the linear fitting of equation (17) yields a slope $m_{Rsh}=0$, a resonance is found at some value of *V*, and just the slightest variation in $I_{sat}$ causes the plot of equation (17) to show large negative or positive values for some values of *V*, causing $m_{Rsh}$ to drastically fluctuate between negative and positive values, making it impossible to find a proper value of $I_{sat}$ that can be considered the single value that causes $m_{Rsh}=0$. In order to describe this, a program was done, called CycleAroot where this was attempted. A video example (named also CycleAroot) of the use of this program can be found in the supplementary material [33], where it is shown the impossibility to use the same idea as in CycleB to find the right value of $I_{sat}$. In this video, an ideal (noiseless) *IV* curve is used to expose this situation. The situation is even worse when *IV* curves with noise are used. Further discussion about this is given below in Section 3 and 4.

Due to this problematic, another program (called CycleAmanual) was done using Procedure A, where the user manually finds the optimal value of $I_{sat}$ that causes $m_{Rsh}=0$. An example of the use of this program can be found in the video called CycleAmanual, in the supplementary material [33]. It is this program that is used in all sections in this article.

All programs simulated the *IV* curves using the deduced parameters for each cycle and equation (2) in [14]. These simulations were used to calculate the integral percentage error (equation (17) in [2]) for each cycle, and the percentage errors. These data, together with the deduced parameters for each cycle, were saved in separate files. You can see video examples of the programs working in the supplementary material as CycleAroot, CycleAmanual and CycleB [33].

The iterative cycles were tested on ideal (noiseless) *IV* curves. These simulations were done on the [0 V, 1 V] range, using the same C program reported in [2]. Laboratory fabricated solar cells usually function in this voltage range [14], [34], [35], [36], [37], [38], [39], [40], [41], [42], [43], [44], [45], [46], [47], [48], [49], [50], [51], [52], [53], [54]. Briefly, the C program solved equation 1 in [2] within an accuracy of 10^−15^ A for each voltage. For simplicity purposes and to facilitate comparison with the deduced solar parameters, all simulation parameters (except *n*) were chosen as power of 10, i.e., the simulation parameters were $R_{sh}=1\;\text{k}\Omega$, $R_{s}=1\;\Omega$, $I_{\mathrm{lig}}=1$ mA and $I_{\mathrm{sat}}=1$ μA. Both $R_{sh}$ and $I_{\mathrm{lig}}$ are three orders of magnitude larger than $R_{s}$ and $I_{\mathrm{sat}}$, respectively, which is usually the case found in second generation solar cells [14], [34], [35], [36], [37], [38], [39], [40], [41], [42], [43], [44], [45], [46], [47], [48], [49], [50], [51], [52], [53], [54]. The value of *n* was chosen n=2.5, which is a value in the range of 2 to 3, which is also the usual case found in second generation solar cells [14], [34], [35], [36], [37], [38], [39], [40], [41], [42], [43], [44], [45], [46], [47], [48], [49], [50], [51], [52], [53], [54]. Values of $P_{V}$ of 11, 21, 51 and 101 $\frac{measured\;points}{V}$ were tested, to obtain the optimal $P_{V}$ that yields reasonable solar cell parameters.

In the following analysis, it was found that $I_{\mathrm{lig}}$ never changed in more than 0.1% from the correct value of 1 mA when recalculating it during the cycles. Then, its evolution during the application of the cycles is not exposed.

It is worth mentioning that currently the suitability of the cycles to deduce the solar cell parameters are currently being investigated for cases of $\frac{R_{s}}{R_{sh}}$ values of 0.01 and 0.1, i.e., with $R_{s}$ values of 10 Ω and 100 Ω, while keeping $R_{sh}=1\;\text{k}\Omega$, and percentage noise of $p_{n}$ =0.01%, 0.1% and 1%. Also, the use of more terms of the polynomial approximation in equation (13) is explored to improve the cycles. The results of this research will be published elsewhere.

The results obtained in this Part 1, are used in real experimental *IV* curves in Part 2 [55], revealing the suitability of the iterative cycles to obtain the solar cell parameters for different solar cells/panels, both in darkness and under illumination [55].

## 3 Analysis using $P_{V}=11\frac{\text{measured points}}{V}$

**Figure 2:**
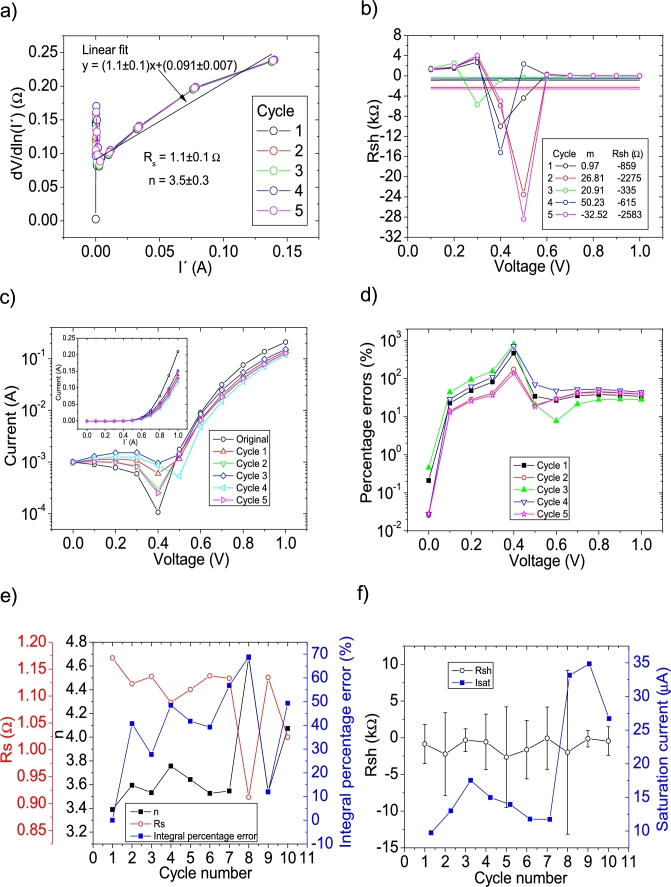
Five cycles application of program CycleAmanual to the *IV* curves and the respective cycles step shown as (a) linear fit of $\frac{\partial V}{\partial lnI^{\prime }}=nkT+R_{s}(I^{\prime })$ vs. *I*′, (b) plot of *R*_*sh*_*vs. V* varying *I*_*sat*_, for the first five cycles, to obtain a horizontal linear fit. The horizontal black, red, green, blue and magenta lines are the linear fitting for Cycles 1 to 5, respectively in (b). The value m is the slope of each linear fit, while the *R*_*sh*_ in the table is the obtained constant of the linear fitting. (c) Logarithm plot of the absolute current vs *V* of the original *IV* curve (in black) and for each resimulations done with the deduced solar cell parameters for each cycle. The same data are plot in the inset as *IV*. (d) Percentage errors between the original *IV* curve and each resimulation shown in (c). (e) Deduced *R*_*s*_ (red), *n* (black) and integral percentage errors (blue) for each cycle. For clarity purposes, the error bars were removed. (f) Deduced *R*_*sh*_ (black) and *I*_*sat*_ (blue) for each cycle.

**Figure 3:**
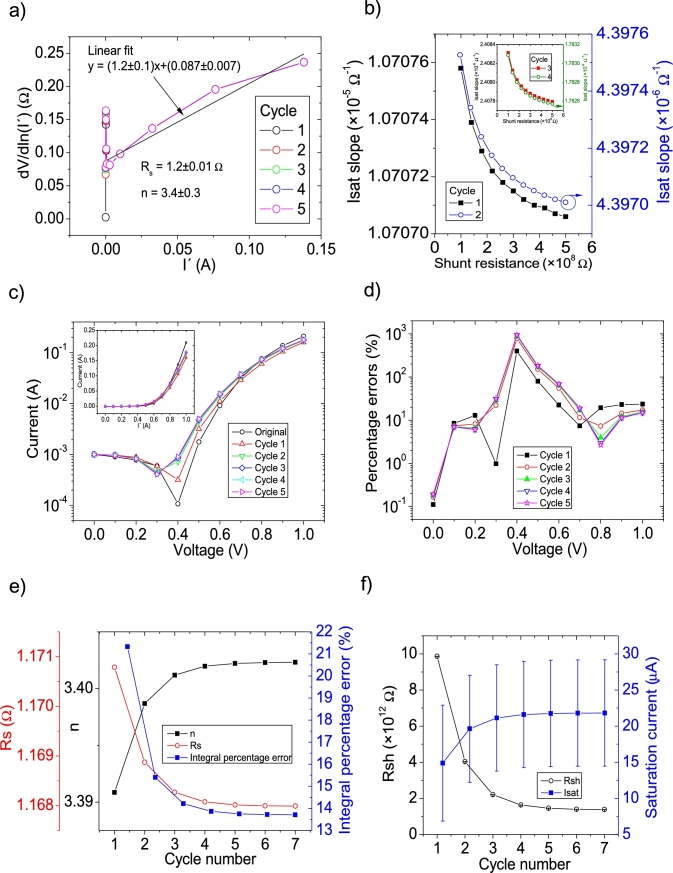
Seven cycles application of program CycleB to the *IV* curves and the respective cycles steps shown as (a) linear fit of $\frac{\partial V}{\partial lnI^{\prime }}=nkT+R_{s}(I^{\prime })$ vs. *I*′, (b) plot of *m*_*Isat*_*vs. R*_*sh*_. (c) Logarithm plot of the absolute current vs *V* of the original *IV* curve (in black) and for each resimulations done with the deduced solar cell parameters for each cycle. The same data are plot in the inset as *IV*. (d) Percentage errors between the original *IV* curve and each resimulation shown in (c). (e) Deduced *R*_*s*_ (red), *n* (black) and integral percentage errors (blue) for each cycle. For clarity purposes, the error bars were removed. (f) Deduced *R*_*sh*_ (black) and *I*_*sat*_ (blue) for each cycle.

**Figure 4:**
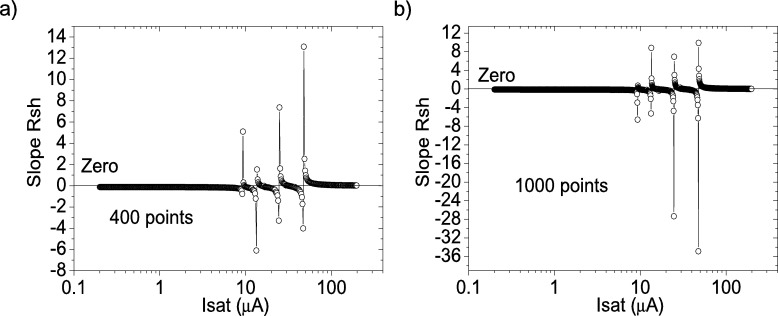
Application of program CycleAroot, with (a) 400 points and (b) 1000 points.

The *IV* curves with $P_{V}=$ 11 $\frac{measured\;points}{V}$ were tested using CycleAmanual and CycleB and the results can be observed in Fig. 2.abcdef) and Fig. 3.abcdef), respectively. Also, CycleAroot was used and its application is seen in Fig. 4.

In both cases, the linear fit of $\frac{\partial V}{\partial lnI^{\prime }}$ *vs.* $I^{\prime }$ yields a value around $R_{s}=1.15\;\Omega$, in reasonable agreement with the original value of $R_{s}=1\;\Omega$. However, a value of n=3.5 is obtained, with an error of 40% from the correct value of n=2.5. The application of Procedure A in CycleAmanual (see Fig. 2(a)) always yielded unrealistic negative values for $R_{sh}$, while $I_{\mathrm{sat}}$ oscillated between 10 and 35 μA. Nevertheless, this did not have a noticeable effect on linear fit of $\frac{\partial V}{\partial lnI^{\prime }}$ *vs.* $I^{\prime }$, when recalculating $I^{\prime }$. As can be seen in Fig. 2(e) and (f), convergence was never achieved using CycleAmanual in any of the solar cell parameters. This is not the case using CycleB, where convergence was achieved in all parameters (see Fig. 3.ef)). Interestingly in this case, *n* converge, but increasing its value from 3.39 to 3.41. This is not the case when $P_{V}$ has larger values, as it is discussed below. The percentage errors using CycleAmanual are around 70%, while using CycleB are around 20% (see Figure 2, Figure 3.d)). Regarding the integral percentage errors, there is no convergence using CycleAmanual, fluctuating between values of 10 and 70% (see Fig. 2.e)). This is not the case using CycleB, where the integral percentage errors converge from a value of 21.5% to 13.5% (see Fig. 3.e)). Further analysis and discussion is given in Section 7.

The application of program CycleAroot, using 400 points and 1000 points in the interval from 0.02 μA to 200 μA, is shown in Fig. 4.ab), respectively. It can be observed that four roots are available, making it impossible to assign one single root. This situation of several roots was also observed when $P_{V}=21\frac{\mathrm{measured}\;\mathrm{points}}{V}$, and it is discussed further in the following Section.

## 4 Analysis using $P_{V}=21\frac{\text{measured points}}{V}$

**Figure 5:**
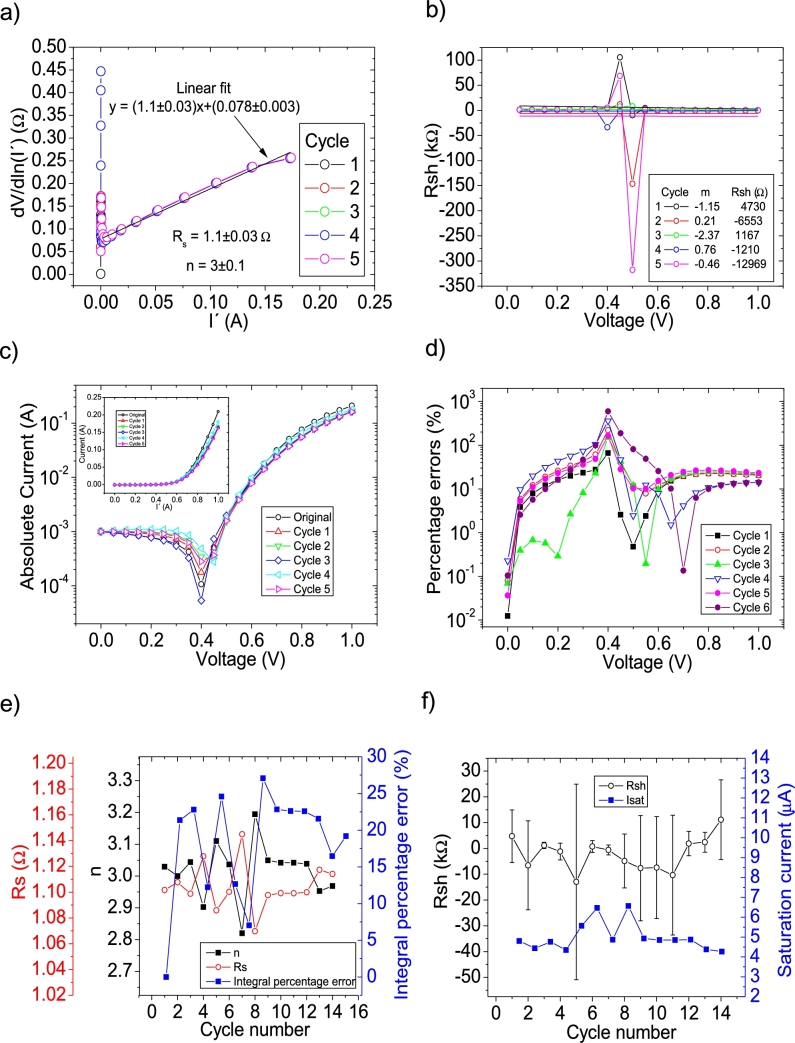
Five cycles application of program CycleAmanual to the *IV* curves and the respective cycles step shown as (a) linear fit of $\frac{\partial V}{\partial lnI^{\prime }}=nkT+R_{s}(I^{\prime })$ vs. *I*′, (b) plot of *R*_*sh*_*vs. V* varying *I*_*sat*_ for each cycle to obtain a horizontal linear fit. The horizontal black, red, green, blue and magenta lines are the linear fitting for Cycles 1 to 5, respectively. The value m is the slope of each linear fit, while the *R*_*sh*_ in the table is the obtained constant of the linear fitting. (c) Logarithm plot of the absolute current vs *V* of the original *IV* curve (in black) and for each resimulations done with the deduced solar cell parameters for each cycle. The same data are plot in the inset as *IV*. (d) Percentage errors between the original *IV* curve and each resimulation shown in (c). (e) Deduced *R*_*s*_ (red), *n* (black) and integral percentage errors (blue) for each cycle. For clarity purposes, the error bars were removed. (f) Deduced *R*_*sh*_ (black) and *I*_*sat*_ (blue) for each cycle.

**Figure 6:**
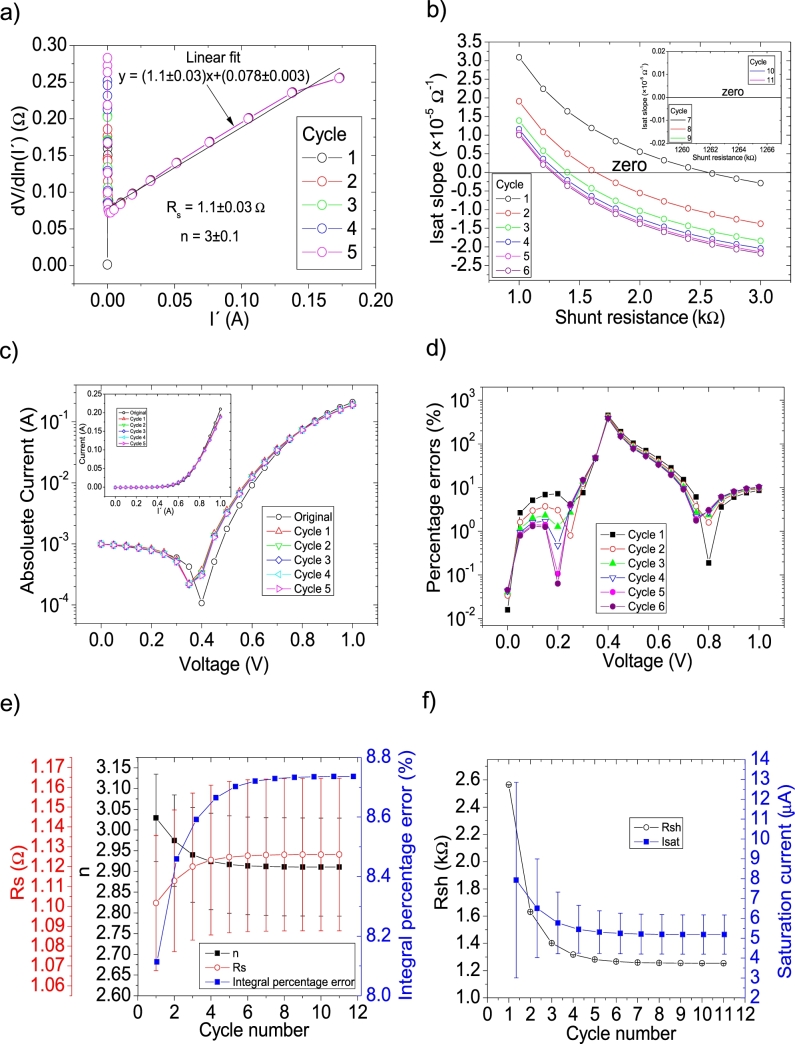
Eleven cycles application of program CycleB to the *IV* curves and the respective cycles steps shown as (a) linear fit of $\frac{\partial V}{\partial lnI^{\prime }}=nkT+R_{s}(I^{\prime })$ vs. *I*′, (b) plot of *m*_*Isat*_*vs. R*_*sh*_. (c) Logarithm plot of the absolute current vs *V* of the original *IV* curve (in black) and for each resimulations done with the deduced solar cell parameters for each cycle. The same data are plot in the inset as *IV*. (d) Percentage errors between the original *IV* curve and each resimulation shown in (c). (e) Deduced *R*_*s*_ (red), *n* (black) and integral percentage errors (blue) for each cycle. (f) Deduced *R*_*sh*_ (black) and *I*_*sat*_ (blue) for each cycle.

**Figure 7:**
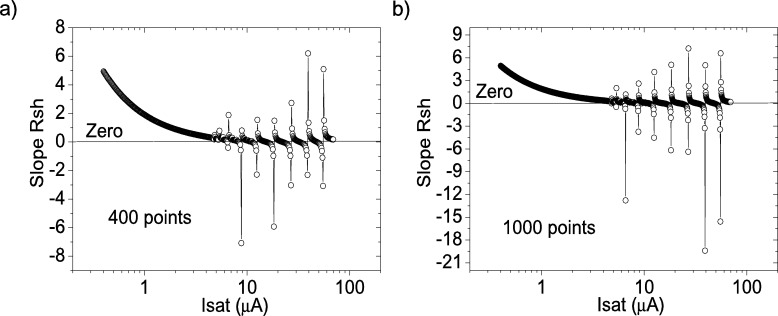
Application of program CycleAroot, with (a) 400 points and (b) 1000 points.

The *IV* curves with $P_{V}=21\frac{measured\;points}{V}$ were tested using CycleAmanual and CycleB and the results can be observed in Fig. 5.abcdef) (Fig. 6.abcdef)), respectively. Also CycleAroot was used and its application is seen in Fig. 7.

In both cases, the linear fit of $\frac{\partial V}{\partial lnI^{\prime }}$ *vs.* $I^{\prime }$ yields a $R_{s}=1.1\;\Omega$, in reasonable agreement with the original value of $R_{s}=1\;\Omega$. In the case of the ideality factor, a value of n=3 is obtained, with an error of 20% from the correct value of n=2.5. The application of Procedure A in CycleAmanual (see Fig. 2.a)) caused $R_{sh}$ to fluctuate between unrealistic negative and positive values, while $I_{\mathrm{sat}}$ oscillated between 4 and 7 μA. In both cases, it never converged, despite fourteen cycles were attempted. Nevertheless, this did not have a noticeable effect on linear fit of $\frac{\partial V}{\partial lnI^{\prime }}$ *vs.* $I^{\prime }$, when recalculating $I^{\prime }$. As can be seen in Fig. 5.ef), convergence was never achieved using CycleAmanual in any of the solar cell parameters. This is not the case using CycleB, where convergence was achieved in all parameters (see Fig. 6.ef)). Contrary to the behaviour shown for $P_{V}=11\frac{measured\;points}{V}$ (see Section 3), *n* converge, but decreasing its value from 3.05 to 2.9. The percentage errors using CycleAmanual are around 30%, while using CycleB are around 10% (see Figure 5, Figure 6.d)). Regarding the integral percentage errors, there is no convergence using CycleAmanual, fluctuating between values of 5 and 25% (see Fig. 2.e)). This is not the case using CycleB, where the integral percentage errors converge, increasing from a value of 8.1% to 8.8% (see Fig. 6.e)). Further analysis and discussion is given in Section 7.

The application of program CycleAroot, using 400 points and 1000 points in the interval from 0.02 μA to 200 μA, is shown in Fig. 7.ab), respectively. It can be observed that at least eight roots are available, causing it impossible to assign one single root. The situation is even worse when increasing the value of $P_{V}$ (not shown in this article). This is the deficiency of program CycleAroot: that a single accurate value of $I_{\mathrm{sat}}$ cannot be determined, and the reason why CycleAmanual was implemented. The application of the program CycleAroot was not investigated further.

## 5 Analysis using $P_{V}=51\frac{\text{measured points}}{V}$

**Figure 8:**
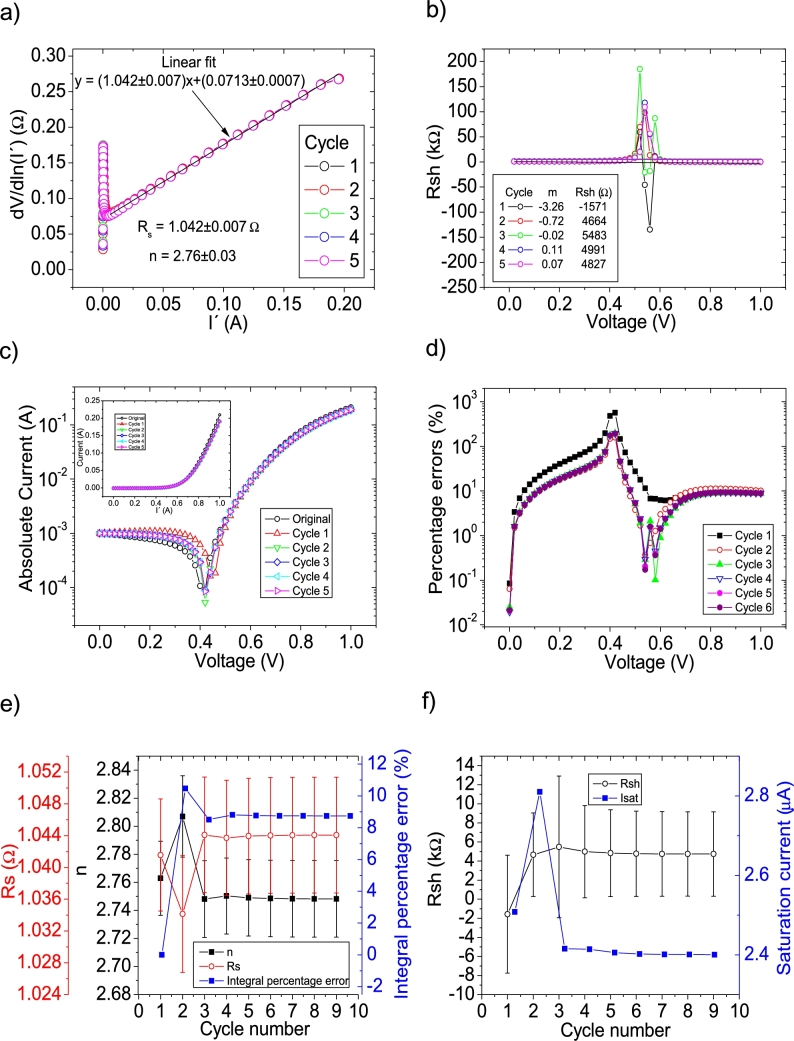
Five cycles application of program CycleAmanual to the *IV* curves and the respective cycles step shown as (a) linear fit of $\frac{\partial V}{\partial lnI^{\prime }}=nkT+R_{s}(I^{\prime })$*vs. I*′, (b) plot of *R*_*sh*_*vs. V* varying *I*_*sat*_ for each cycle to obtain a horizontal linear vit. The horizontal black, red, green, blue and magenta lines are the linear fitting for Cycles 1 to 5, respectively. The value m is the slope of each linear fit, while the *R*_*sh*_ in the table is the obtained constant of the linear fitting. (c) Logarithm plot of the absolute current vs *V* of the original *IV* curve (in black) and for each resimulations done with the deduced solar cell parameters for each cycle. The same data are plot in the inset as *IV*. (d) Percentage errors between the original *IV* curve and each resimulation shown in (c). (e) Deduced *R*_*s*_ (red), *n* (black) and integral percentage errors (blue) for each cycle. For clarity purposes, the error bars were removed. (f) Deduced *R*_*sh*_ (black) and *I*_*sat*_ (blue) for each cycle.

**Figure 9:**
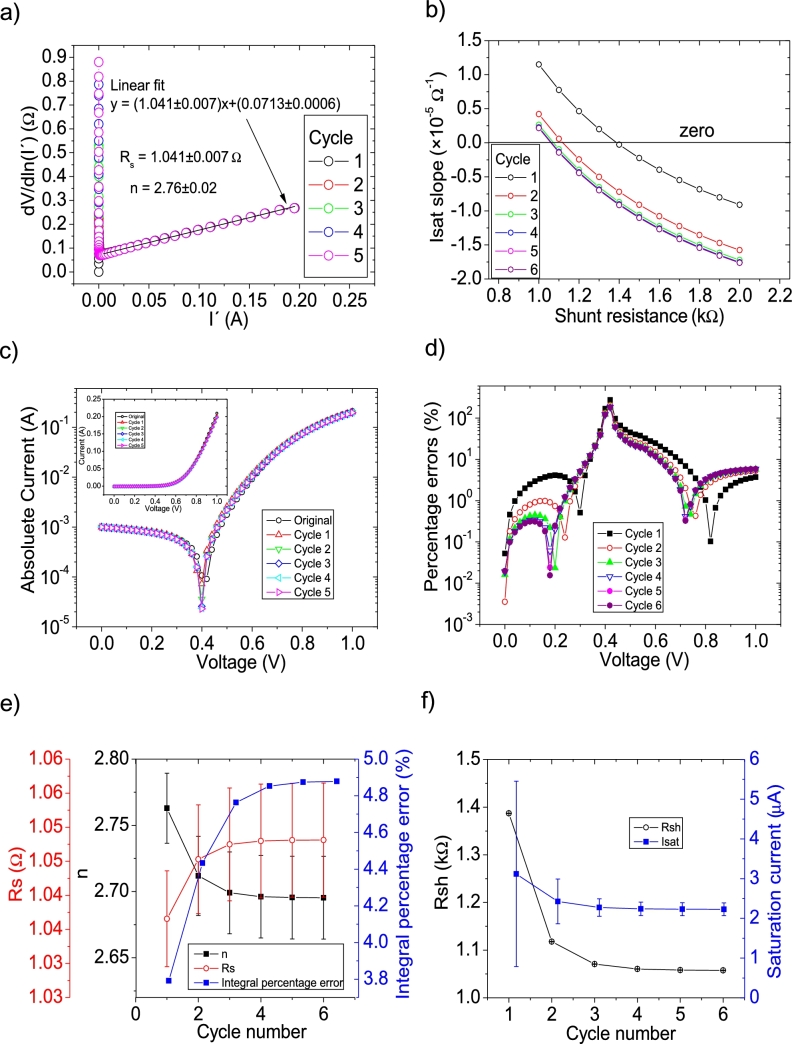
Six cycles application of program CycleB to the *IV* curves and the respective cycles steps shown as (a) linear fit of $\frac{\partial V}{\partial lnI^{\prime }}=nkT+R_{s}(I^{\prime })$*vs. I*′, (b) plot of *m*_*Isat*_*vs. R*_*sh*_. (c) Logarithm plot of the absolute current vs *V* of the original *IV* curve (in black) and for each resimulations done with the deduced solar cell parameters for each cycle. The same data are plot in the inset as *IV*. (d) Percentage errors between the original *IV* curve and each resimulation shown in (c). (e) Deduced *R*_*s*_ (red), *n* (black) and integral percentage errors (blue) for each cycle. (f) Deduced *R*_*sh*_ (black) and *I*_*sat*_ (blue) for each cycle.

The *IV* curves with $P_{V}=51\frac{measured\;points}{V}$ were tested using CycleAmanual and CycleB and the results can be observed in Fig. 8.abcdef) (Fig. 9.abcdef)), respectively.

In both cases, the linear fit of $\frac{\partial V}{\partial lnI^{\prime }}$ *vs.* $I^{\prime }$ yields a $R_{s}=1.04\;\Omega$, in reasonable agreement with the original value of $R_{s}=1\;\Omega$. In the case of the ideality factor, a value of n=2.76 is obtained, with an error of 10 % from the correct value of n=2.5. The solar cell parameters converge to reasonable values, using both CycleB and CycleAmanual (see Fig. 8.ef) and Fig. 9.ef)). However, all of them fluctuate during the first three cycles. Six cycles are a reasonable number of cycles to achieve convergence. The percentage errors using CycleAmanual are around 50% (10%) for low (high) voltages, while using CycleB are around 10% (see Figure 8, Figure 9.d)). Regarding the integral percentage errors, using CycleAmanual, it quickly converges to a value of 9% (see Fig. 8.e)). This is not the case using CycleB, where the integral percentage errors converge, increasing from a value of 3.8% to 4.9% (see Fig. 9.e)). Further analysis and discussion is given in Section 7.

## 6 Analysis using $P_{V}=101\frac{\text{measured points}}{V}$

**Figure 10:**
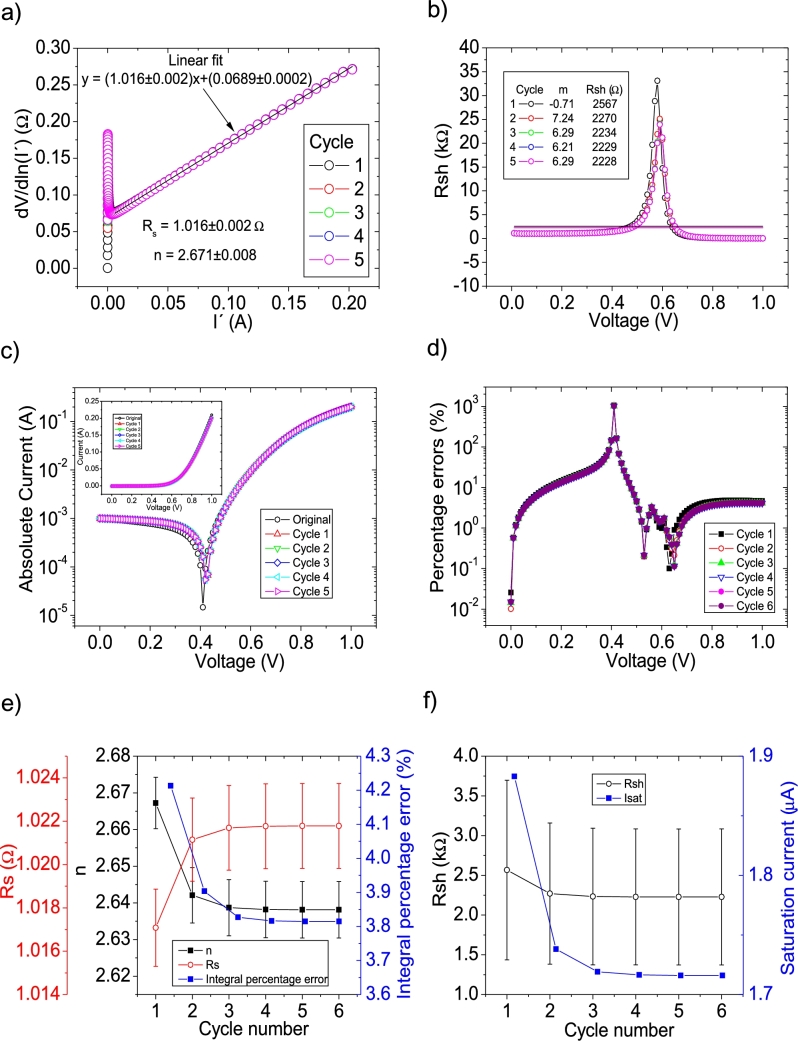
Five cycles application of program CycleAmanual to the *IV* curves and the respective cycles step shown as (a) linear fit of $\frac{\partial V}{\partial lnI^{\prime }}=nkT+R_{s}(I^{\prime })$ vs. *I*′, (b) plot of *R*_*sh*_*vs. V* varying *I*_*sat*_ for each cycle to obtain a horizontal linear fit. The horizontal black, red, green, blue and magenta lines are the linear fitting for Cycles 1 to 5, respectively. The value m is the slope of each linear fit, while the *R*_*sh*_ in the table is the obtained constant of the linear fitting. (c) Logarithm plot of the absolute current vs *V* of the original *IV* curve (in black) and for each resimulations done with the deduced solar cell parameters for each cycle. The same data are plot in the inset as *IV*. (d) Percentage errors between the original *IV* curve and each resimulation shown in (c). (e) Deduced *R*_*s*_ (red), *n* (black) and integral percentage errors (blue) for each cycle. For clarity purposes, the error bars were removed. (f) Deduced *R*_*sh*_ (black) and *I*_*sat*_ (blue) for each cycle.

**Figure 11:**
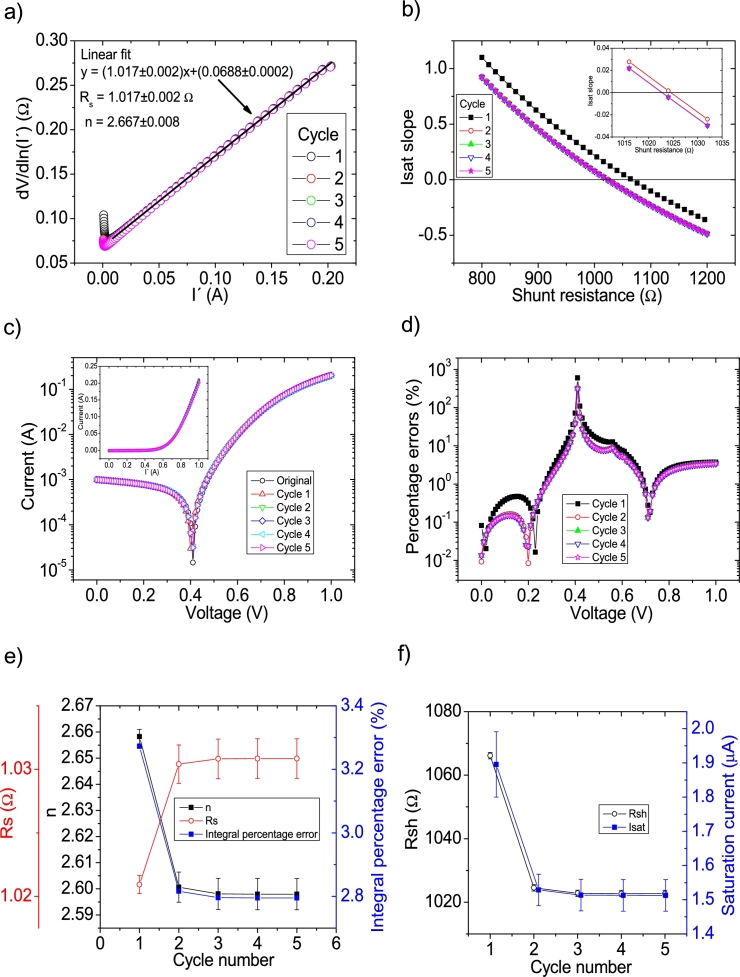
Five cycles application of program CycleB to the *IV* curves and the respective cycles steps shown as (a) linear fit of $\frac{\partial V}{\partial lnI^{\prime }}=nkT+R_{s}(I^{\prime })$ vs. *I*′, (b) plot of *m*_*Isat*_*vs. R*_*sh*_. (c) Logarithm plot of the absolute current vs *V* of the original *IV* curve (in black) and for each resimulations done with the deduced solar cell parameters for each cycle. The same data are plot in the inset as *IV*. (d) Percentage errors between the original *IV* curve and each resimulation shown in (c). (e) Deduced *R*_*s*_ (red), *n* (black) and integral percentage errors (blue) for each cycle. (f) Deduced *R*_*sh*_ (black) and *I*_*sat*_ (blue) for each cycle.

The *IV* curves with $P_{V}=101\frac{measured\;points}{V}$ were tested using CycleAmanual and CycleB and the results can be observed in Fig. 10.abcdef) (Fig. 11.abcdef)), respectively.

In both cases, the linear fit of $\frac{\partial V}{\partial lnI^{\prime }}$ *vs.* $I^{\prime }$ yields a $R_{s}=1.01\;\Omega$, in reasonable agreement with the original value of $R_{s}=1\;\Omega$. In the case of the ideality factor, a value of n=2.67 is obtained, with an error of 6.8 % from the correct value of n=2.5. The solar cell parameters converge to reasonable values, using both CycleAmanual and CycleB (see Fig. 10.ef) and Fig. 11.ef)). Four cycles are a reasonable number of cycles to achieve convergence. The percentage errors using CycleAmanual are around 10% (5 %) for low (high) voltages, while using CycleB are around 5% (see Figure 10, Figure 11(d)). Regarding the integral percentage errors, using CycleAmanual (CycleB), it quickly converges to a value of 3.8% (2.8%) (see Fig. 10.e) and Fig. 11.e)). Further analysis and discussion is given in Section 7.

## 7 Application of the Ortiz-Conde et al. and the Zhang et al. method

**Figure 12:**
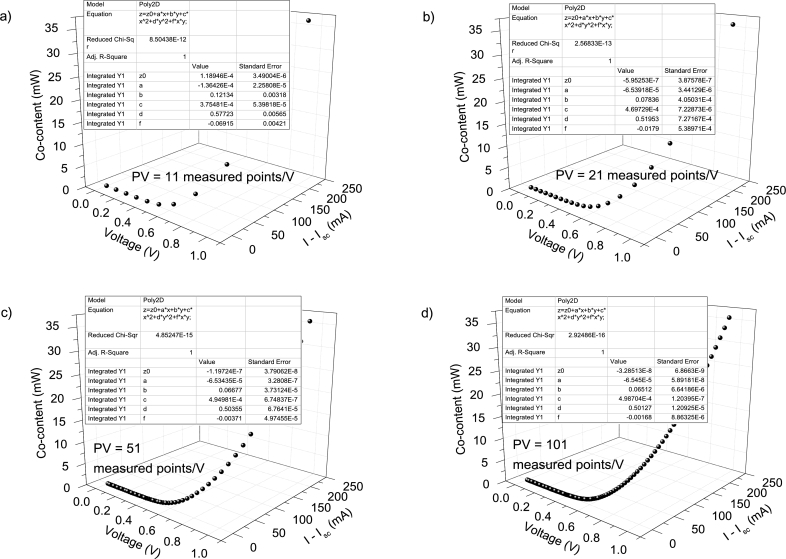
Application of the Ortiz-Conde *et al.* method [14] to (a) IV1, (b) IV2, (c) IV3 and (d) IV4.

The Ortiz-Conde *et al.* technique [14] and the Zhang *et al.* technique [28], are two common techniques used to obtain the solar cell parameters. A brief discussion about them can be found in Section 3 of [16]. In Fig. 12.abcd)-13, the application of Ortiz-Conde *et al.* technique [14] is shown, while in Fig. 14.ab), the application of Zhang *et al.* technique [28] is exposed, in IV4. The MATLAB optimization procedure proposed by Zhang *et al.* was not applied, which is the reason why $I_{\mathrm{sat}}$ was not obtained in this case [28]. Their results, together with the results of the application CycleAmanual and CycleBroot, are summarized in Table 1. In Fig. 15.abcd), the percentage errors obtained by each method, relative to the original known values of $R_{sh}=1\;\text{k}\Omega$, $R_{s}=1\;\Omega$, $I_{\mathrm{sat}}=1$ μA, and n=2.5, are given, as function of $P_{V}$ of 11, 21, 51 and 101 $\frac{measured\;points}{V}$.

**Figure 13:**
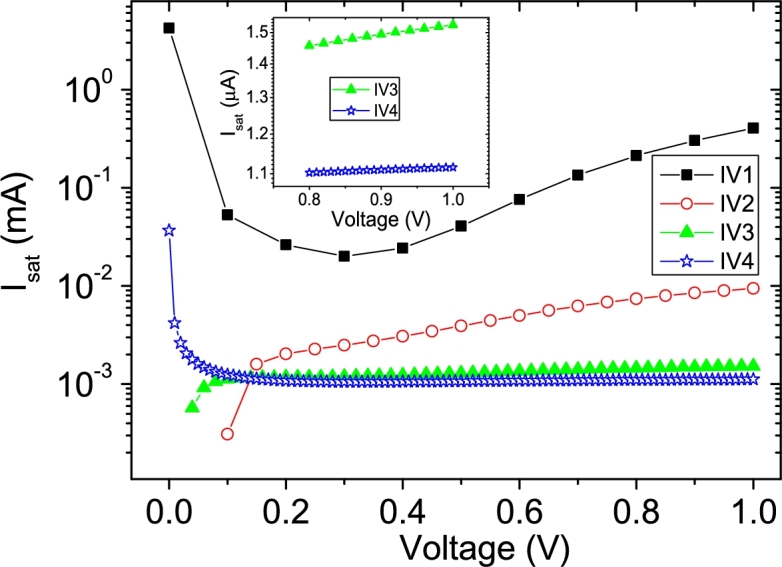
Plot of *I*_*sat*_ according to Eq. 10 in [2]. In the inset, the voltage range [0.8 V, 1 V] for IV3 and IV4 is shown, revealing that no convergence has been achieved yet.

**Figure 14:**
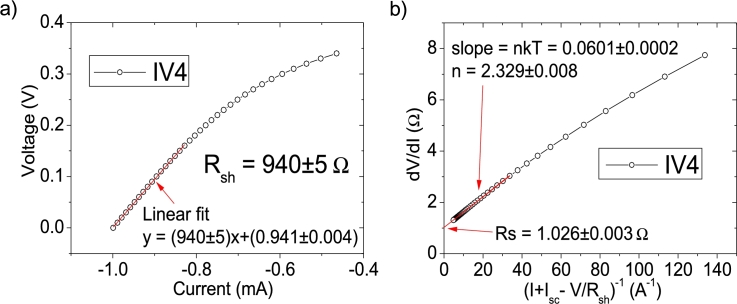
Application of the Zhang *et al.*[28] method on IV4.

**Table 1:** Results of the application of the Ortiz-Conde et al. [14] (no superscript), Zhang et al. techniques [28] (superscript (b)), CycleA (superscript (c)), and CycleB (superscript (d)).

Curve	C_I1_	C_V1_	C_I2_	C_V2_
IV1	0.121±0.003	(−1.4±0.2)×10^−4^	0.577±0.006	(3.8±0.5)×10^−4^
IV2	0.0784±0.0004	(−6.5±0.3)×10^−5^	0.5195±0.0007	(4.7±0.07)×10^−4^
IV3	0.06677±0.00003	(−6.53±0.03)×10^−5^	0.50355±0.00007	(4.95±0.006)×10^−4^
IV4	0.06512±0.000006	(−6.55±0.006)×10^−5^	0.50127±0.00001	(4.99±0.001)×10^−4^


	*R*_*sh*_ (Ω)	*R*_*s*_ (Ω)	*n*	*I*_*lig*_ (mA)	*I*_*sat*_ (μA)
IV1	1332±2×10^−11^	1.15±0.02	4.7±0.1	1.04±0.04	402.775
	^*b*^ 737±72	^*b*^ 1.17±0.09	^*b*^ 2.24±0.04	^*b*^ 0.998	
	^*c*^ unrealistic	^*c*^ 1.2±0.09	^*c*^ 4.1±0.3	^*c*^ 0.991	^*c*^ 26.7±1×10^−10^
	^*d*^ (1.4±0.0002)×10^12^	^*d*^ 1.2±0.1	^*d*^ 3.4±0.3	^*d*^ 0.999	^*d*^ 22±7
IV2	1064±3×10^−12^	1.038±0.003	3.03±0.02	0.991±0.005	9.44604
	^*b*^ 847±32	^*b*^ 1.06±0.02	^*b*^ 2.29±0.02	^*b*^ 0.998	
	^*c*^ unrealistic	^*c*^ 1.11±0.04	^*c*^ 2.97±0.1	^*c*^ 1	^*c*^ 4.2698±0.0001
	^*d*^ 1253.6±0.5	^*d*^ 1.12±0.04	^*d*^ 2.91±0.1	^*d*^ 1	^*d*^ 5.2±0.9
IV3	1010±3×10^−13^	1.0061±0.0002	2.585±0.001	0.9992±0.0005	1.52524
	^*b*^ 911±11	^*b*^ 1.028±0.004	^*b*^ 2.337±0.008	^*b*^ 0.998	
	^*c*^ 4744.6±4424	^*c*^ 1.044±0.007	^*c*^ 2.75±0.03	^*c*^ 0.999	^*c*^ 2.4006±0.0001
	^*d*^ 1057±0.5	^*d*^ 1.053±0.008	^*d*^ 2.7±0.03	^*d*^ 1	^*d*^ 2.2±0.2
IV4	1003±5×10^−14^	1.00154±0.00005	2.5215±0.0003	1.00055±0.00008	1.11568
	^*b*^ 940±5	^*b*^ 1.026±0.003	^*b*^2.329±0.008	^*b*^ 0.998	
	^*c*^ 2228±855	^*c*^ 1.022±0.002	^*c*^ 2.63±0.008	^*c*^ 0.999	^*c*^ 1.7161±0.0001
	^*d*^ 1022.7±0.5	^*d*^ 1.031±0.002	^*d*^ 2.598±0.006	^*d*^ 1	^*d*^ 1.51±0.05

**Figure 15:**
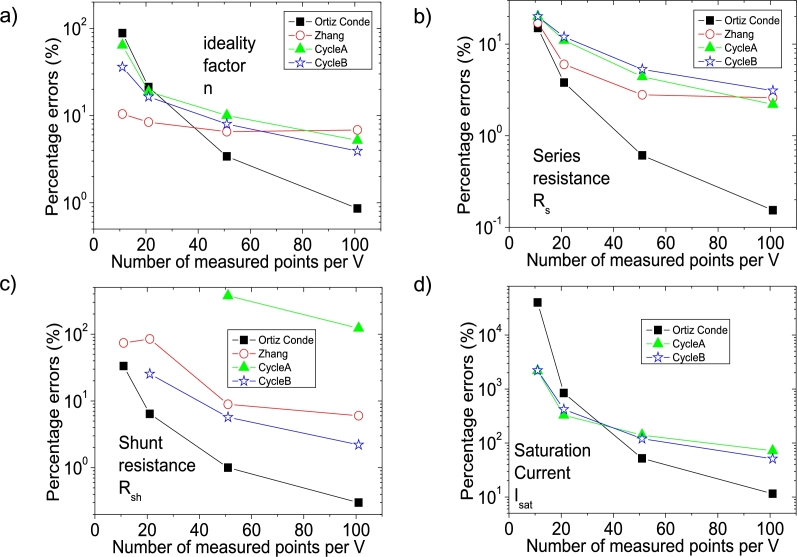
Percentage errors calculated according to Table 1, respect the known original values of (a) *n* = 2.5, (b) *R*_*s*_ = 1 Ω, (c) *R*_*sh*_ = 1 kΩ and (d) *I*_*sat*_ = 1 μA.

## 8 Discussion

CycleAmanual and CycleB yield similar percentage errors for *n* and $R_{s}$, compared to the Ortiz-Conde *et al.* method [14] for points $P_{V}=11\frac{measured\;points}{V}$, but larger than the Zhang *et al.* method [28]. The percentage errors rapidly decrease as $P_{V}$ increases, decreasing faster when using the Ortiz-Conde *et al.* method [14], while the percentage errors obtained using the Zhang *et al.* method [28] decrease when $P_{V}$ increases to $21\frac{measured\;points}{V}$, not changing substantially if $P_{V}$ increases further. Regarding $R_{sh}$, CycleAmanual and CycleB have the disadvantage of yielding larger values than those obtained using the Ortiz-Conde *et al.* method [14], but still CycleAmanual provide more accurate values than the Zhang *et al.* method [28]. However, the impact of overestimation of $R_{sh}$ is negligible when simulating the curves (see Figure 2, Figure 3, Figure 4, Figure 5, Figure 6, Figure 7, Figure 8, Figure 9, Figure 10, Figure 11). Concerning $I_{\mathrm{sat}}$, both CycleAmanual and CycleB yield more accurate values than those provided by the Ortiz-Conde *et al.* method $P_{V}=21\frac{measured\;points}{V}$ or smaller, and relatively similar in case $P_{V}=51\;\frac{measured\;points}{V}$. In the case $P_{V}=101\frac{measured\;points}{V}$, provide more accurate value of $I_{\mathrm{sat}}$. It is worth mentioning that the smaller percentage errors in general obtained using the Ortiz-Conde *et al.* method [14] could be an artifact of the ideality (noiseless) nature of the *IV* curves. In fact, as it is discussed in [13], [15], [16], once the *IV* curves are real measured *IV* curves (including noise), the accuracy of the Ortiz-Conde *et al.* method [14] strongly depend both on the percentage noise and $P_{V}$, and unrealistic negative solar cell parameters can be obtained, when the percentage noise is as low as 0.01% [13], [15]. It is currently investigated how accurate are CycleAmanual and CycleB as function of percentage noise, and it will be reported elsewhere.

Comparing CycleAmanual and CycleB among them, reasonable and similar values of $R_{s}$ are obtained when doing the linear fit of $\frac{\partial V}{\partial lnI^{\prime }}$ *vs.* $I^{\prime }$, independently of the value of $P_{V}$. Regarding the value of *n*, it improves as the value of $P_{V}$ increases, also independently if it is program CycleAmanual and CycleB which is used.

Regarding the extraction of $R_{sh}$, and $I_{\mathrm{sat}}$, a minimum value of $P_{V}=$ 51 $\frac{measured\;points}{V}$ is necessary to achieve convergence, in case program CycleAmanual is used. This is not the case when using CycleB, which obtains reasonable values of $R_{sh}$, and $I_{\mathrm{sat}}$ for values of $P_{V}=$ 11 $\frac{measured\;points}{V}$, and reasonably reproduce the original *IV* curve.

The percentage errors and integral percentage errors, are always larger when using CycleAmanual than CycleB, showing that CycleB extracts more accurately the solar cell parameters. It is worth mentioning, that the program automatically yields these percentage errors.

Finally, the number of cycles necessary to achieve convergence diminishes as the value of $P_{V}$ increases, as expectable.

## 9 Conclusion

Two iterative cycles, namely CycleA and CycleB have been proposed. They are based on: 1) the linear fit of $\frac{\partial V}{\partial lnI^{\prime }}$ *vs.* $I^{\prime }$, where $I^{\prime }=I+I_{sat}+I_{lig}-\frac{V-IR_{s}}{R_{sh}}+\frac{nkT}{R_{sh}}$, which yields $R_{s}$ and *n*, 2) the application of Procedure A or Procedure B [2] for CycleA or CycleB, respectively, to obtain $I_{\mathrm{sat}}$ and $R_{sh}$, 3) the correction of $I_{\mathrm{lig}}$ once $R_{s}$, *n*, $I_{\mathrm{sat}}$ and $R_{sh}$, and 4) the repetition of the cycles till convergence is obtained.

CycleB was implemented as one single program, where the user applied Procedure B plotting $m_{Isat}$ *vs.* $R_{sh}$ and finding the suitable root. CycleA was implemented as two programs. The first one, called CycleAroot, a similar idea of plotting $m_{Rsh}$ *vs.* $I_{sat}$ and finding the suitable root was attempted. It was found that this plot has several roots, and it is not possible to choose one single of them. Then, the second implemented program, namely CycleAmanual, was implemented, where the user manually finds the proper value of $I_{sat}$ that causes $m_{Rsh}=0$.

The results exposed in this article show that reasonable solar cell parameter extraction is achieved when using these cycles. It was found that CycleB yields more accurate solar cell parameter extraction than any of the CycleA programs, independently of the value of $P_{V}$. In the case of CycleA, a minimum value of $P_{V}=$ 51 $\frac{measured\;points}{V}$ is necessary to achieve proper solar cell parameter extraction.

## Declarations

### Author contribution statement

Victor Tapio Rangel Kuoppa: Conceived and designed the experiments; Performed the experiments; Analyzed and interpreted the data; Contributed reagents, materials, analysis tools or data; Wrote the paper.

### Funding statement

Victor Tapio Rangel Kuoppa was supported by the 10.13039/100010897Newton Fund (RCUK-CONACyT 2016 FONCICYT/68).

### Data availability statement

Data will be made available on request.

### Declaration of interest's statement

The authors declare no conflict of interest.

### Additional information

Supplementary content related to this article has been published online at https://doi.org/10.1016/j.heliyon.2022.e10551.

## Appendix A Supplementary material

CycleAmanual correct.mp4 Video explaining the use of CycleAmanual. CycleAmanual correct.mp4

CycleAroot correct.mp4 Video explaining the use of CycleAroot. CycleAroot correct.mp4

CycleB correct.mp4 Video explaining the use of CycleB. CycleB correct.mp4
